# Prof. White’s Return

**Published:** 1851-12

**Authors:** 


*Prof. White's return.* — Prof. J. P. White returned in the steamer Franklin, on the 2d ult., after an absence of ten months, in excellent health, and has resumed his active duties as a practitioner and teacher. He has added to his means of illustrating his course on obstetrics, an extensive and valuable collection of preparations, models, etc., selected by himself during his sojourn in Europe.

